# Retinal Thickness and the Structure/Function Relationship in the Eyes of Older Adults with Glaucoma

**DOI:** 10.1371/journal.pone.0141293

**Published:** 2015-10-27

**Authors:** Megumi Honjo, Kazuko Omodaka, Tatsuro Ishizaki, Shinji Ohkubo, Makoto Araie, Toru Nakazawa

**Affiliations:** 1 Department of Ophthalmology, the University of Tokyo Graduate School of medicine, Tokyo, Japan; 2 Department of Ophthalmology, Tokyo Metropolitan Geriatric Hospital, Tokyo, Japan; 3 Department of Ophthalmology, Tohoku University Graduate School of Medicine, Miyagi, Japan; 4 Longitudinal Interdisciplinary Study on Aging, Tokyo Metropolitan Institute of Gerontology, Tokyo, Japan; 5 Department of Ophthalmology and Visual Science, Kanazawa University Graduate School of Medical Science, Kanazawa, Japan; 6 Kanto Central Hospital, Tokyo, Japan; Universidade Federal do Rio de Janeiro, BRAZIL

## Abstract

Glaucoma is common and shows high prevalence in older adults. However, there are few studies on the structure/function relationship in older adults with glaucoma. This prospective, cross-sectional study (conducted between February and August 2014), enrolled 102 eyes of 102 subjects aged over 75 years, including 57 eyes with primary open angle glaucoma (POAG), 15 eyes with pseudoexfoliation glaucoma (PXG), and 30 healthy eyes. Multiple regression analysis was used to determine the correlation of circumpapillary retinal nerve fiber layer thickness (cpRNFLT) and macular parameters to mean deviation (MD) to and standard automated perimetry (SAP)-measured sensitivity, assessed with the 30–2 and 10–2 programs. In each 10–2 SAP test point, Spearman’s rank correlation coefficient was used to compare macular retinal nerve fiber layer thickness (mRNFLT), macular ganglion cell-inner plexiform layer thickness (GCIPLT), and mRNFL+GCIPL thickness (GCCT) with sensitivity after adjusting for retinal ganglion cell (RGC) displacement. In eyes with POAG and PXG, cpRNFLT was significantly correlated with 30–2 MD and 30–2 sensitivity. Multiple regression analysis revealed that the POAG had significantly lower cpRNFLT, mRNFLT, GCIPLT, and GCCT according to the severity of disease than control eyes after adjusting for sensitivity, age, sex, and axial length. The PXG eyes had significantly lower cpRNFLT, mRNFLT, and GCCT when compared with the early to moderate POAG eyes. GCCT was significantly correlated with 10–2 sensitivity, except in one juxtafoveal point, (r = 0.338–0.778) in the POAG eyes. The periphery of the central 10° area showed a good correlation between sensitivity and mRNFLT, while the central 5.8° showed a good correlation between sensitivity and GCIPLT. The correlation between structure and function was significant, and objective and quantitative method with OCT assessing glaucoma that does not require patient ability could be a possible parameter to assess diagnosis and progression in older patients with glaucoma.

## Introduction

Glaucoma is a leading cause of irreversible blindness worldwide [1]. Despite the high prevalence of glaucoma in older adults [2], no studies have examined the relationship between the structure of the eye and its function in older individuals with glaucoma, especially in those aged 75 years or more.

The recent development of spectral-domain optical coherence tomography (SD-OCT) allows us to precisely and easily measure retinal nerve fiber layer (RNFL) thickness (RNFLT). The loss of RNFLT has been reported to often precede the onset of glaucomatous visual field (VF) loss [3], and has been shown to be a reliable way of detecting glaucoma, although standard automated perimetry (SAP) remains the standard method of diagnosing glaucoma [4]. However, in older adults, SAP measurements do not always have sufficient reliability [5]. Evaluations of the severity and progression of glaucoma in older individuals can be inadequate when they rely solely on SAP measurements, which can be affected by poor patient performance. Additionally, the image quality of acquired images tends to become poorer with age, due to both impaired patient collaboration and the increasing prevalence of opacities of the optic media. Therefore, older patients may particularly benefit from the development of an objective method to assess glaucoma.

A number of studies have used SD-OCT to specifically examine the relationship between structural and functional glaucomatous damage. These studies have demonstrated that VF sensitivity is highly correlated to circumpapillary RNFLT (cpRNFLT), ganglion cell-inner plexiform layer (GCIPL) thickness (GCIPLT), and macular ganglion cell complex (GCC) thickness (GCCT; a combination of macular RNFL thickness (mRNFLT) and GCIPLT) [6–10]. Additional investigation has demonstrated that while global VF sensitivity is also highly correlated to cpRNFLT [11], the strength of the structure/function relationship in eyes with glaucoma depends on the severity of the disease [12], [13]. Several groups have reported that the location of macular inner retinal thickness loss correlated well with the location of macular sensitivity loss in glaucoma [14–18]. We have previously reported that GCCT was the most useful parameter to evaluate the relationship between the structure and function of the central 10° of eyes with primary open angle glaucoma (POAG), and found that adjusting for RGC displacement was essential to effectively evaluate the GCL-related layers [19]. However, our study, like other previous studies, included only relatively younger individuals [19], [20]. No study has yet provided a precise description of the structure/function relationship in the eyes of older adults with glaucoma or the effects of aging on this relationship, despite the reported influence of age on SAP sensitivity [21], [22] and the reported age-related decrease in the OCT-determined thickness of retinal layers such as the GCIPL and GCC [23–25]. In the present study, we therefore assessed the localized structure/function relationship in older adults with glaucoma.

## Materials and Methods

### Participants

This prospective, cross-sectional study was conducted at Tokyo Metropolitan Geriatric Hospital. Only individuals aged over 75 years were recruited. The Institutional Review Board and Ethics Committee of the Tokyo Metropolitan Geriatric Hospital and Institute of Gerontology approved the study, which also adhered to the tenets of the Declaration of Helsinki.

Glaucoma patients who had undergone at least three VF tests and age-matched self-reported healthy volunteers were enrolled in this study between February 2014 and August 2014. Healthy subjects were recruited from the general population of outpatients at Tokyo Metropolitan Geriatric Hospital. All participants with written informed consent underwent an ocular examination including auto-refractometer measurement, best-corrected visual acuity (BCVA) measurement, slit-lamp examination, axial length measurement with the IOL Master (Carl Zeiss Meditec Inc), intraocular pressure (IOP) measurement, dilated fundoscopy, visual field testing with the Humphrey 30–2 Swedish Interactive Threshold Algorithm (SITA) Fast Strategy (SAP-F) (Carl Zeiss Meditec Inc) and the Humphrey 10–2 SITA Standard Strategy (SAP-S), and SD-OCT examination with a 3D-OCT 2000 (Topcon Corp., Tokyo, Japan). In our experience, the reliability of SAP measurement in older adults (aged over 75 years) with glaucoma can be affected by a long measurement time (unpublished data). As this is especially true for the 30–2 SAP-S program, we chose the SITA Fast Strategy when performing the 30–2 SAP test (30–2 SAP-F). All examinations were conducted within 3 months of an SD-OCT examination. The exclusion criteria were: unreliable visual field measurements, an axial length > 26.0 mm or < 21.0 mm; any ocular or systemic diseases that might affect visual field or the optic nerve. Cataracts have been reported to significantly affect SD-OCT measurements of RNFLT in regard to signal strength [26], [27]. All subjects in this study were therefore pseudophakic without clinically significant posterior capsule opacities. If both eyes of a subject were eligible for inclusion, the eye with worse mean deviation (MD) in 10–2 SAP-S testing was selected.

The POAG and PXG groups included patients with glaucomatous VF defects that were confirmed in at least 2 reliable VF examinations, using the Anderson and Patella criteria [28]. PXG was diagnosed by the presence of exfoliated material, ascertained before the cataract surgery. The eyes with POAG were classified into groups based on the severity of the disease: early POAG (30–2 SAP-F MD ≥ -6dB), moderate POAG (-6dB > 30–2 SAP-F MD ≥ -12dB), and severe POAG (-12dB > 30–2 SAP-F MD). Exclusion criteria for the normal group were, IOP 22 mmHg or higher, BCVA worse than 0.1 (logMAR), VF defects suggestive of glaucoma according to the Anderson-Patella criteria [28], and any abnormal VF loss consistent with ocular or systemic diseases.

### OCT examination

SD-OCT examinations were performed as previously described [19], [20]. An experienced examiner (MH) confirmed the validity of the image segmentation. Only images with a quality factor > 60 were used in the analysis. Briefly, a macular OCT map corresponding to the 68 10–2 SAP test points was obtained. First, three-dimensional imaging data were obtained from a macular cube scan of a 7 × 7 mm square area, and a 6 × 6 mm area was selected for analysis. The thickness of the retinal layers at each test point was automatically calculated by the included 3D OCT software. Location adjustment for RGC displacement within the OCT measurement area corresponding to each 10–2 SAP test point was approximated using a single equation (*y* = 1.29 × [*x* + 0.046]^0.67^, *y* = RGC eccentricity, *x* = cone eccentricity), which relates cone and corresponding RGC eccentricity, as defined by Sjöstrand et al [29].

### Visual field analysis

Visual sensitivity and total deviation (TD) at each 10–2 test point were measured in decibels, and were calculated as unlogged 1/Lambert (1/L) values, as follows: the decibel readings were divided by 10 and the quotient was then unlogged [19]. Visual fields measured with the 10–2 program were classified as abnormal, as in previous reports [30].

### Statistical analysis

Data were discarded if the scan quality did not satisfy the criteria described above. Statistical analysis was performed with SPSS software version 20.0 (SPSS, Inc., Chicago, IL, USA). An analysis of variance (ANOVA) and chi-square test were used. We evaluated the correlation between VF parameters and OCT parameters with Spearman’s rank correlation coefficient and a multiple linear regression analysis. A p-value < 0.05 was considered statistically significant, unless otherwise noted.

## Results

**Fig 1:**
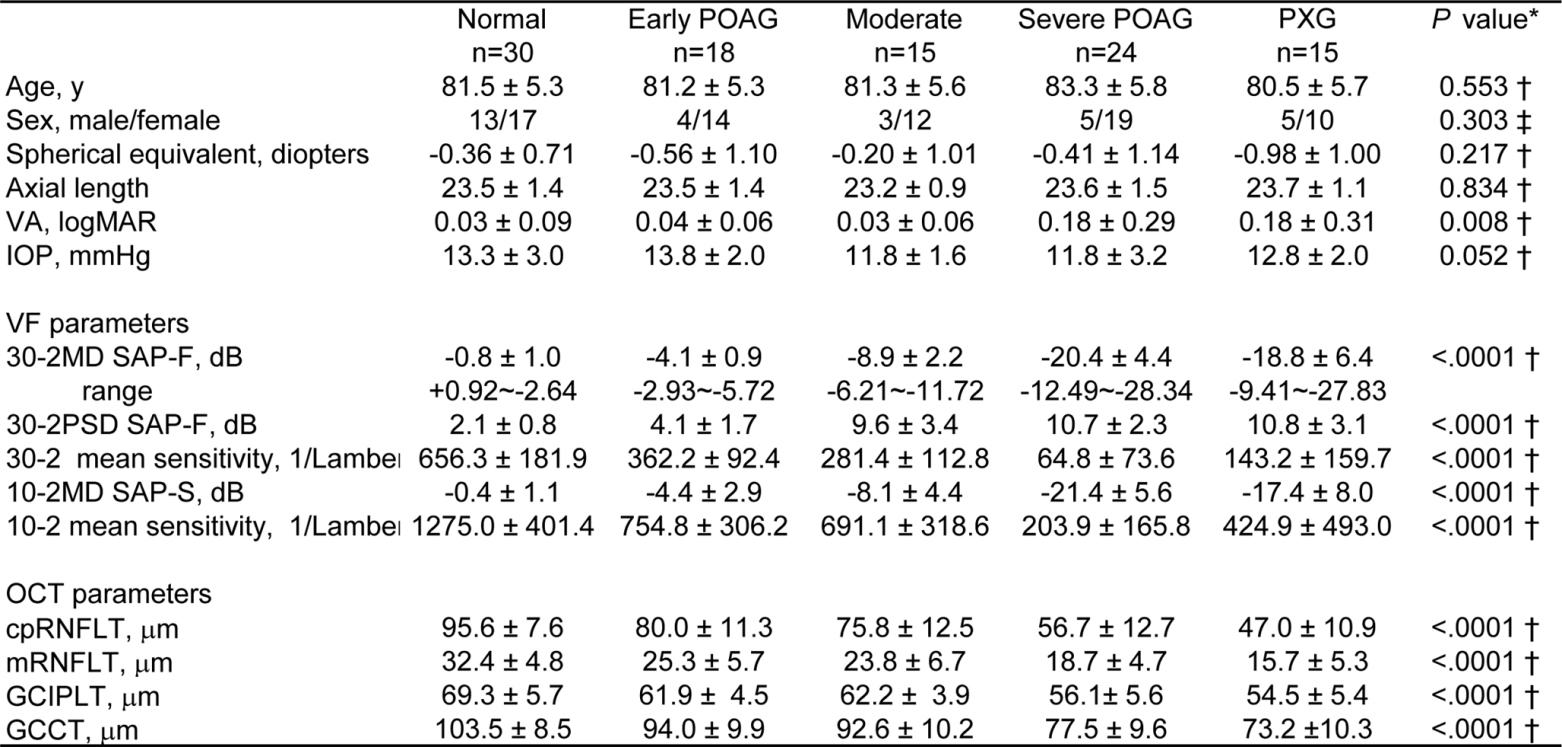
Characteristics of the 102 study subjects, visual field parameters, and OCT parameters. Values correspond to mean ± standard deviation unless noted otherwise. * Difference between normal and glaucoma. †ANOVA ‡ Chi-square test.

This study included a single eye of each of 102 subjects, all aged 75 years or older, comprising 72 eyes with glaucoma and 30 healthy eyes. The eyes with glaucoma included 57 with POAG and 15 with PXG (Fig 1). During the enrollment period, we examined 139 eyes of glaucoma patients from whom we could obtain OCT images with a quality factor > 60. Sixty-two eyes were excluded because of unreliable SAP measurements (30–2: n = 33; 10–2: n = 29). Two eyes were excluded because of an epiretinal membrane that resulted in poor-quality images. Three eyes in which an erroneous RNFL or GCC profile of 0.0 μm was computed, as a result of poor delineation, were excluded from the analysis (RNFL: n = 1; GCC: n = 2).

**Fig 2:**
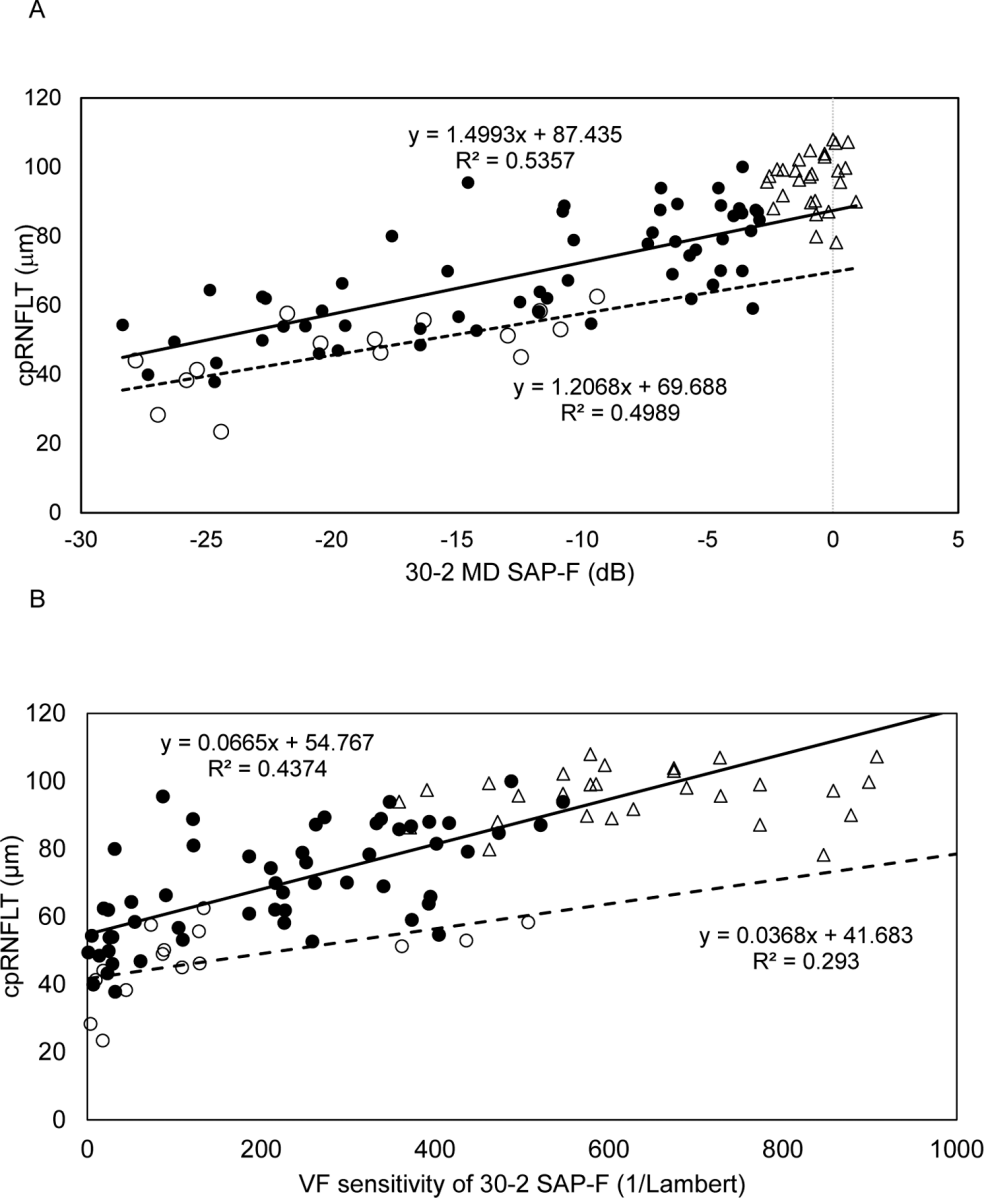
Scatter plot graph showing the correlation of optical coherence tomography-measured circumpapillary retinal nerve fiber layer thickness to 30–2 SAP-F-measured mean deviation (dB) (A) and 30–2 SAP-F-measured VF sensitivity (1/Lambert) (B), in normal, primary open-angle glaucoma (POAG) and pseudoexfoliation glaucoma (PXG) subjects. The formula used in the linear regression analysis of each subject subtype is also shown. Open triangles indicate normal subject data. Filled circles and an unbroken regression line indicate POAG subjects. Open circles and a dotted regression line indicate PXG subjects.

To find the correlation between cpRNFLT and the VF parameters from the 30–2 SAP-F in all study subjects, a simple regression analysis was performed. Fig 2 shows a scatter plot graph of the correlation of cpRNFLT to 30–2 SAP-F-measured MD (dB) and 30–2 SAP-F-measured VF sensitivity (1/L) in the normal, POAG and PXG subjects. There was a significant correlation between cpRNFLT and 30–2 SAP-F MD in the POAG (r = 0.732, p<0.001) and PXG (r = 0.706, p = 0.003) subjects, but not in the normal subjects (r = 0.067, p = 0.738). We also found a significant correlation between cpRNFLT and 30–2 SAP-F VF sensitivity in the POAG (r = 0.661, p<0.0001) and PXG (r = 0.541, p = 0.037) subjects, but not in the normal subjects (r = 0.032, p = 0.868).

**Fig 3:**
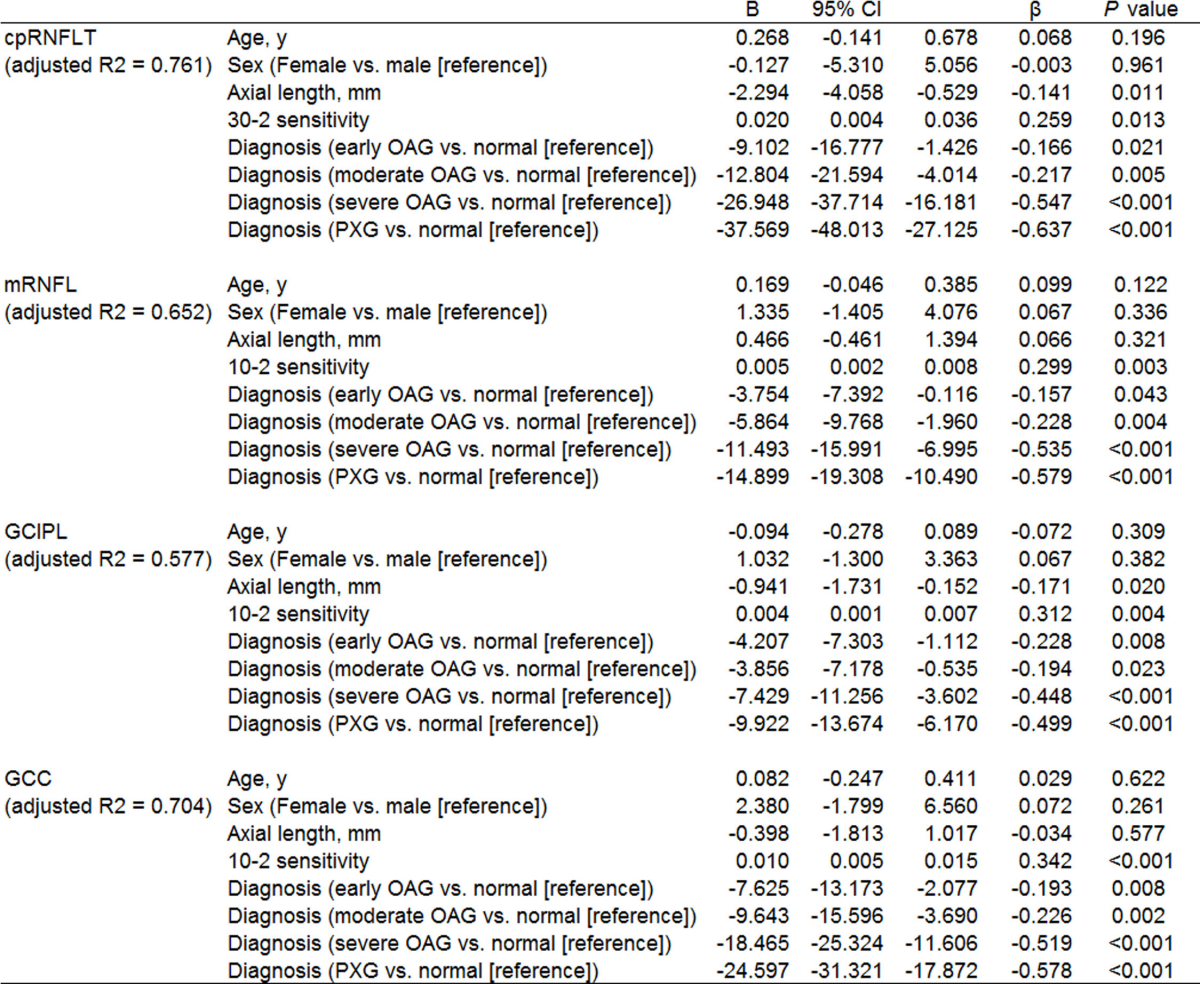
Multiple regression analysis of factors associated with cpRNFLT, mRNFLT, GCIPLT, and GCCT in POAG (n = 57), in PXG (n = 15) and healthy (n = 30) subjects. Either cpRNFLT, mRNFLT, GCIPLT, or GCCT were set as the dependent variable, and age, sex, axial length, 30–2 sensitivity or 10–2 sensitivity, and diagnosis were set as the independent variables. B = partial regression coefficient. 95% CI = 95% confidence interval of partial regression coefficient. β = standardized partial regression coefficient. Adjusted R2 = adjusted coefficient of multiple determination.

**Fig 4:**
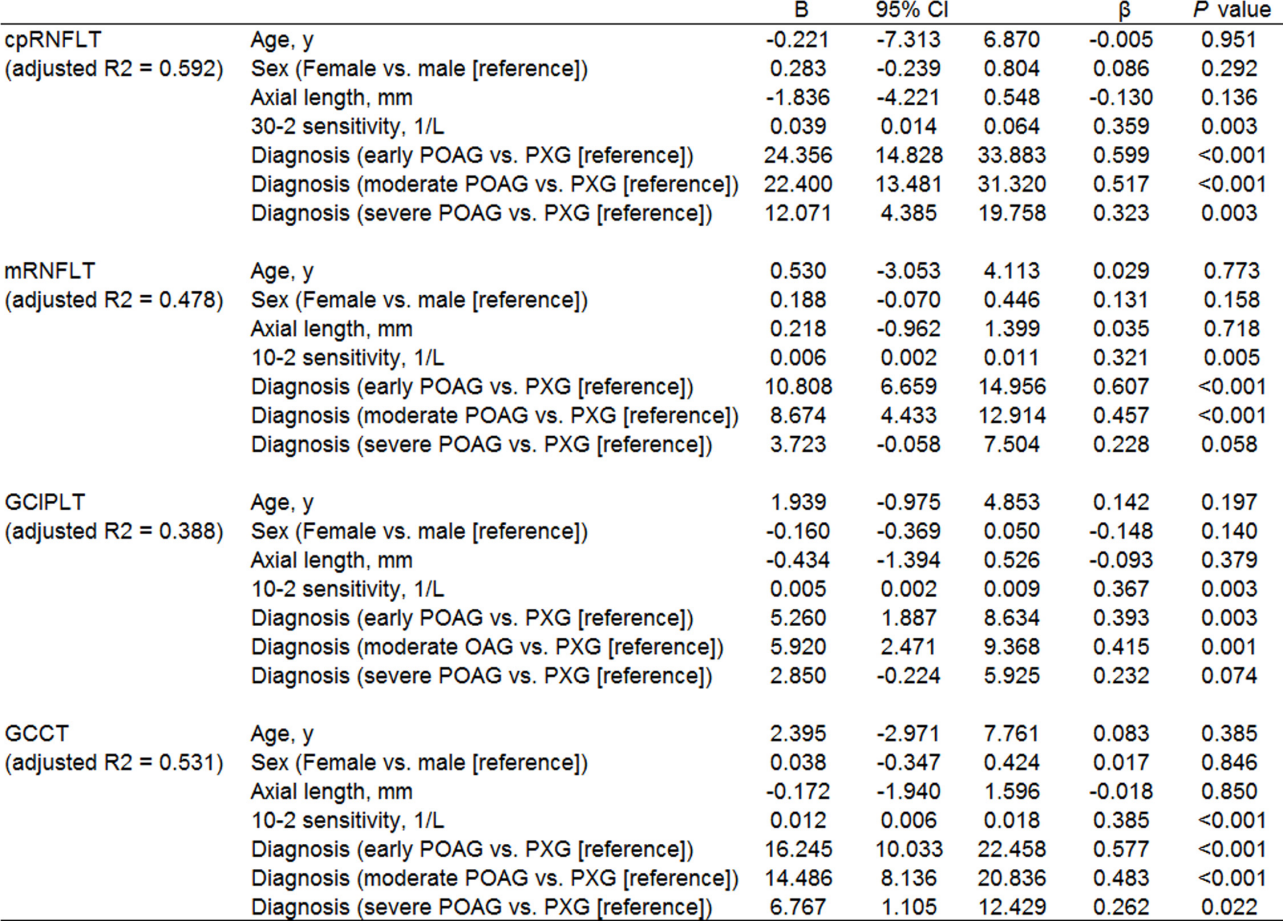
Multiple regression analysis of factors associated with cpRNFLT, mRNFLT, GCIPLT, and GCCT in different stages of POAG (n = 57) and in PXG (n = 15). Either cpRNFLT, mRNFLT, GCIPLT, GCCT were set as the dependent variable, and age, sex, axial length, 30–2 sensitivity or 10–2 sensitivity, and diagnosis were set as the independent variables. B = partial regression coefficient. 95% CI = 95% confidence interval of partial regression coefficient. β = standardized partial regression coefficient. Adjusted R2 = adjusted coefficient of multiple determination.

Fig 1 shows data from OCT measurements in the control and glaucoma groups. As expected, all parameters had high values in the control group and lower values in the glaucoma groups, decreasing with glaucoma severity. Thus, we performed a multiple regression analysis to determine factors associated with cpRNFLT, mRNFLT, GCIPLT and GCCT after adjusting for explanatory variables such as age, sex, axial length, and sensitivity, which have been reported to influence retinal structure [24], [31]. Either cpRNFLT, mRNFL, GCIPL, or GCC were set as the dependent variable, and age, sex, axial length, 30–2 sensitivity or 10–2 sensitivity, and diagnosis were set as the independent variables. cpRNFLT was significantly lower, with partial regression coefficients of -9.102 (p = 0.021), -12.804 (p = 0.005), -26.948 (p<0.001) and -37.569 (p<0.001) respectively, in the early POAG, moderate POAG, severe POAG and PXG patients, with reference to the normal subjects (Fig 3). Analyses of each macular layer yielded essentially the same results: the POAG patients had significantly lower macular thickness than normal controls, progressing with the severity of glaucomatous damage, and PXG patients had significantly lower mRNFLT, GCIPLT and GCCT with reference to normal subjects (Fig 3). Further analysis of factors associated with cpRNFLT, mRNFLT, GCIPLT, and GCCT among patients with PXG (n = 15) and those with POAG (n = 57) revealed that the PXG patients had significantly lower cpRNFLT, mRNFLT, GCIPLT and GCCT. Furthermore, the partial regression coefficient differed with the severity of POAG: with reference to the early (n = 18) or moderate (n = 15) POAG patients, the PXG patients had significantly lower cpRNFLT, mRNFLT, GCIPLT and GCCT, while with reference to the severe POAG patients (n = 24), the partial correlation coefficients were significant for cpRNFLT and GCCT, but not for mRNFLT or GCIPLT (Fig 4).

**Fig 5:**
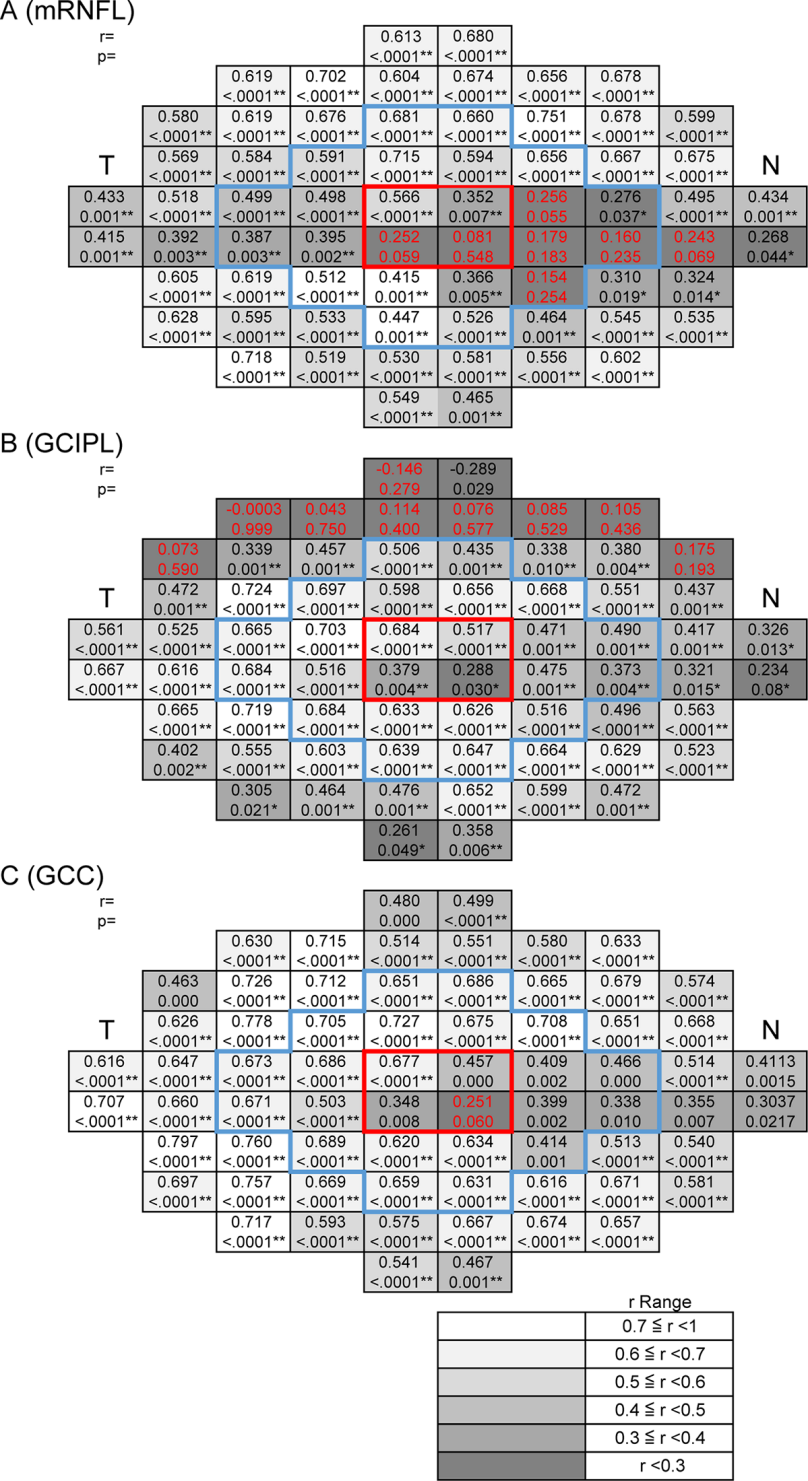
The correlation between visual field sensitivity and macular inner retinal thickness at each test point with retinal ganglion cell (RGC) displacement in primary open-angle glaucoma (POAG) subjects. The correlation was determined between VF sensitivity and mRNFLT, GCIPLT, and GCCT at each test point in the POAG patients, after adjustment for RGC displacement. The grayscale; r ranges are shown at the bottom right. Upper case characters indicate Spearman’s rank correlation coefficients. Lower case characters indicate the P-values of the Spearman’s rank correlation coefficients. T indicates temporal, N indicates nasal. The area surrounded by the red frame shows the 4 central points. The area surrounded by the blue frame shows the area within 5.8°. * indicates P < 0.05, ** indicates P <0.01. Red letters indicate that the correlation was not significant. (A) Macular retinal nerve fiber layer (mRNFL), (B) ganglion cell layer + inner plexiform layer (GCIPL), (C) mRNFL + GCIPL (GCC).

We further investigated the correlation between VF sensitivity and mRNFLT, GCIPLT, and GCCT at each test point in the POAG patients, after adjustment for RGC displacement (Figs 5A–5C). mRNFLT in the POAG patients was significantly correlated with 10–2 sensitivity, except in the papillomacular bundle region, after adjustment for RGC displacement (r = 0.081–0.751) (Fig 5A). A significant correlation of sensitivity to GCIPLT and GCCT was observed after adjustment for RGC displacement. GCIPLT was significantly correlated in all 24 points in the central 5.8° in the POAG patients (r = 0.288–0.703) (Fig 5B). An area of high correlation between sensitivity and mRNFLT was located in the periphery of the central 10°, while an area of high correlation between sensitivity and GCIPLT was found in the central 5.8°.

**Fig 6:**
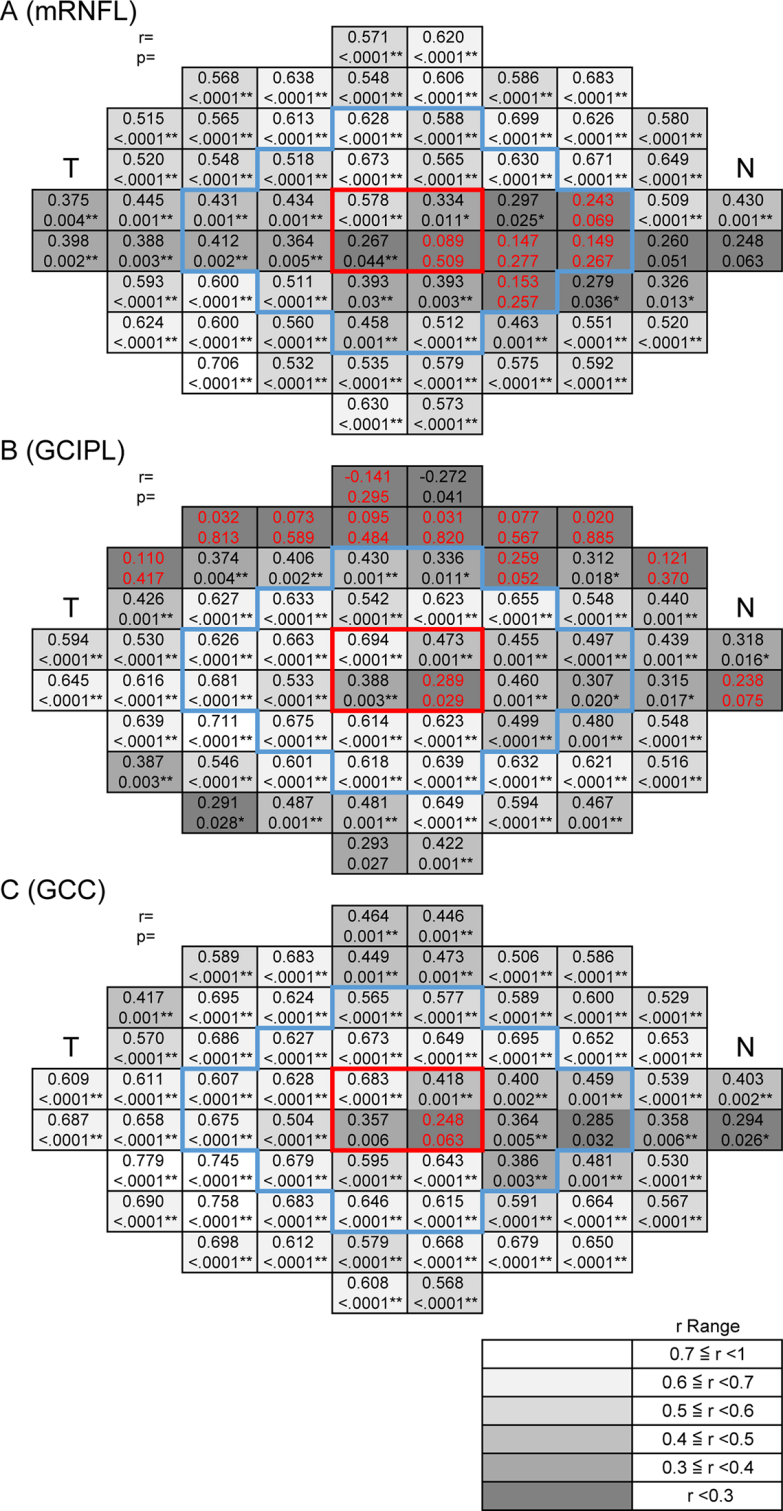
The correlation between total deviation and macular inner retinal thickness at each test point with retinal ganglion cell (RGC) displacement in primary open-angle glaucoma (POAG) subjects. The correlation was determined between total deviation and mRNFLT, GCIPLT, and GCCT at each test point in the POAG patients, after adjustment for RGC displacement. The grayscale; r ranges are shown at the bottom right. Upper case characters indicate Spearman’s rank correlation coefficients. Lower case characters indicate the P-values of the Spearman’s rank correlation coefficients. T indicates temporal, N indicates nasal. The area surrounded by the red frame shows the 4 central points. The area surrounded by the blue frame shows the area within 5.8°. * indicates P < 0.05, ** indicates P <0.01. Red letters indicate that the correlation was not significant. (A) Macular retinal nerve fiber layer (mRNFL), (B) ganglion cell layer + inner plexiform layer (GCIPL), (C) mRNFL + GCIPL (GCC).

An analysis of the correlation between TD and mRNFLT, GCIPLT, and GCCT in the POAG patients yielded essentially the same results (Fig 6). GCCT in the POAG patients was significantly correlated with both sensitivity and TD in almost all the 68 test points of the 10–2 SAP program, except for one juxtafoveal point, after adjustment for RGC displacement (98.5%) (sensitivity: r = 0.348–0.778; TD: r = 0.357–0.745) (Figs 5C and 6C). These results correspond well with our previous findings in younger individuals [19], [20].

**Fig 7:**
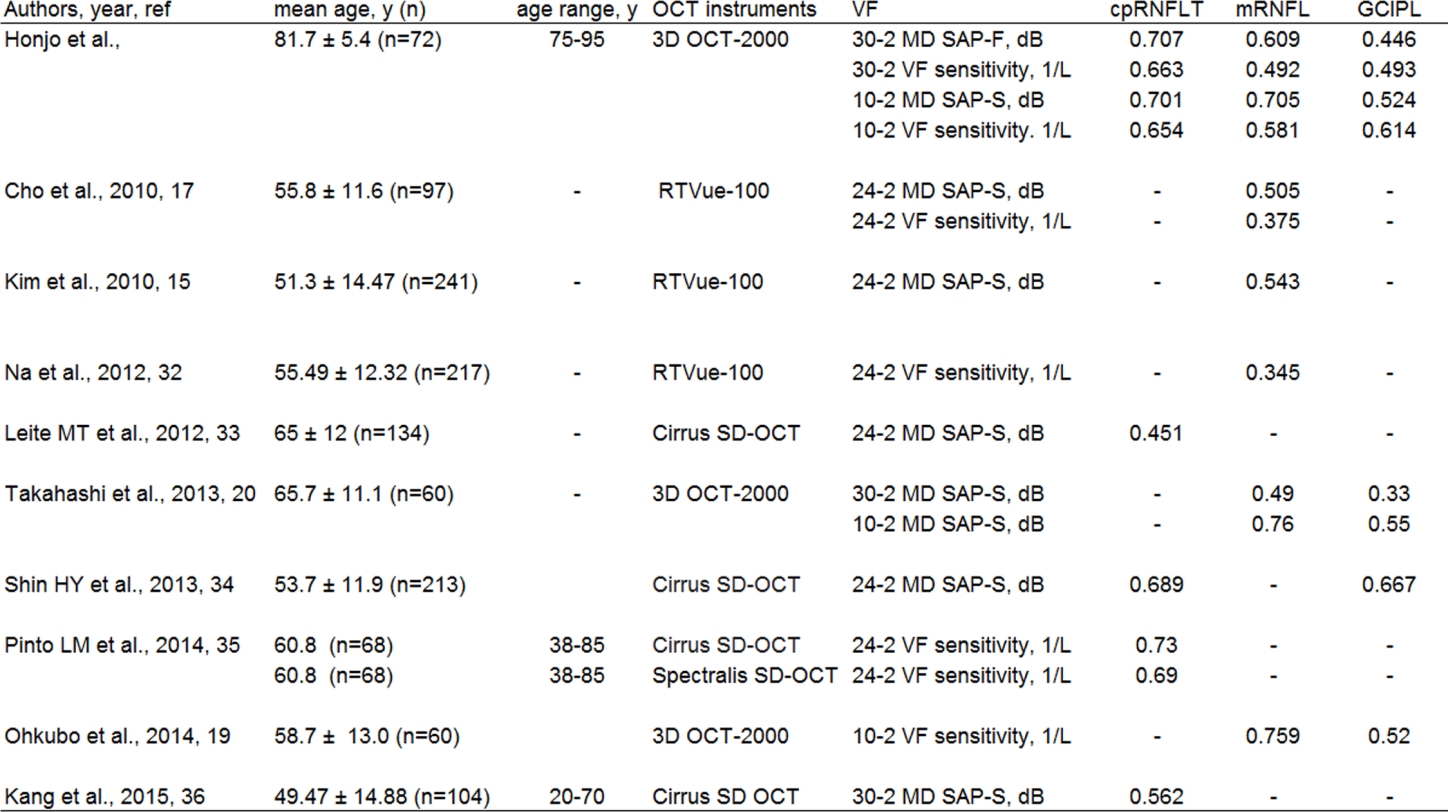
The reported correlation between visual field and OCT parameters in glaucoma subjects [15], [17], [19], [20], [32–36]. Values correspond to mean ± standard deviation unless otherwise noted. SAP-F: Humphrey Swedish Interactive Threshold Algorithm (SITA) Fast Strategy, SAP-S: Humphrey SITA Standard Strategy.

In order to determine whether older glaucoma patients have a similar structure/function correlation as younger patients, we performed a literature search of previously published studies in which a similar evaluation of the structure/function correlation was performed, with the results shown in Fig 7. Although these previous studies had differences in mean age, SAP measurement strategy and OCT instrumentation, their analyses of the structure/function correlation in younger patients agree with the results of our present study, both in the correlation between cpRNFLT and SAP 30–2 and the correlation between macular parameters and SAP 10–2.

## Discussion

New imaging technologies provide promise to allow a better understanding of the relationship between structure and function in eyes with glaucoma, and several recent studies have demonstrated that the high diagnostic potential of cpRNFLT and GCCT [8], [9]. Nevertheless, there are still few specific data on older individuals. In the present study, we therefore examined a group composed solely of individuals aged 75 years or older with glaucoma and investigated the regional correlation between VF measurements and OCT measurements.

It is well known that cpRNFLT decreases rapidly in early-to-moderate glaucoma before reaching a plateau in advanced glaucoma. In the present study, we found that the OCT measurement values were high in the control group and decreased with increasing glaucoma severity in the POAG patients, determined by multiple regression analysis (Figs 1, 3 and 4). We also found that average cpRNFLT and macular thickness to be lower in older individuals, not only in glaucoma patients but also in healthy subjects (Fig 1). It is reported that older individuals show lower cpRNFLT and decrease of SAP sensitivity with age [10], [37–39]. Despite the presence of age-related decrease of both cpRNFLT and SAP sensitivity, the present study revealed that the relationship between cpRNFLT and MD and 30–2 sensitivity was significant in the POAG and PXG eyes in older patients (Fig 2). This relationship was stronger with 30–2 MD (dB) than with sensitivity (1/L), due to the range of VF measurements.

The eyes with PXG had lower OCT parameters measurements than the eyes with POAG (Fig 1), and multiple regression analysis revealed that cpRNFLT and GCCT were significantly lower in the PXG eyes than in the early to severe POAG eyes (Fig 4). PXG is an age-related disease associated with faster progression than POAG [40], [41]. Although it has been reported that PXG patients have lower cpRNFLT and a thinner lamina cribrosa than normal subjects or POAG patients [42–46], little is known about the structure/function relationship in PXG patients. Our study only included limited number of subjects with a limited range of VF sensitivities. Further studies with larger numbers of patients are needed to confirm the significance of thinned OCT measurement parameters in PXG patients.

In the macula, this study showed that sensitivity was significantly correlated to GCIPLT and GCCT within the central 5.8° in POAG eyes (Fig 5). On the other hand, the correlation between sensitivity and mRNFLT was relatively low in the papillomacular bundle region within the central 5.8°, even after adjustment for RGC displacement. However, sensitivity and mRNFLT were highly correlated in the periphery of the central 10°, while sensitivity and GCIPLT were highly correlated in the central area. GCCT was significantly correlated with sensitivity in almost all test points of the 10–2 SAP program, except for one nasal lower juxtafoveal test point, after adjustment for RGC displacement. These results were somewhat consistent with those of previous studies conducted in younger patient populations, but GCIPLT exhibited a worse correlation in both the superior and inferior periphery in previous studies, while the present study found an area of lower correlation with the GCL-related layers only in the superior periphery [19], [20]. We speculate that there may be several reasons for the difference: SAP measurements have been reported to be influenced by age; sensitivity decreases with age [10]; and the influence of age varies across different areas, being greater in the periphery and superior than in the center and in the inferior half of the visual field [47]. In normal eyes, the age-related decrease in GCIPLT beyond the central 6° has been reported to be greater in the inferior macula [18], [48]. In eyes with progressive glaucoma, GCIPLT has been reported to decrease from 50.0% to 14.7% [49]. Finally, the superior macular region is known to be less susceptible to glaucomatous damage than the inferior macular region [10]. Taken together, the poor correlation between sensitivity and GCIPLT in the superior hemifield in older individuals may therefore be at least partially attributable to the combined influence of age-related sensitivity deterioration, thinning of the GCL-related layers and the greater susceptibility of the inferior macula. We have previously reported that age-related thickness loss was slightly lower in the GCC and higher in the GCIPL than we expected from a review of the literature on normal subjects [50]. Compared to the GCC, loss of GCIPL thickness in older glaucoma patients may be unevenly distributed, with a wide range that is affected by factors related to both age and glaucomatous changes. Overall, our results showed that out of all areas of the central 10°, GCCT was the most useful parameter for analyzing the retinal structure/function relationship in older individuals with glaucoma. Further studies will be needed to investigate age-related changes in macular parameters.

To the best of our knowledge, this study, in which the median age of the subjects was over 80 years, is the first report on the correlation between the structure of the retina and SAP sensitivity in older glaucoma patients. Both VF testing and evaluation of structural changes are important in the diagnosis and monitoring of glaucoma patients [51], [52]. However, ageing can affect SAP measurement and lower its reliability index [5]. A study of normal individuals with a mean age of 62 years showed that half had abnormal or unreliable visual fields [53]. Furthermore, many older individuals with glaucoma have difficulty with clinical VF examinations due to cognitive impairment or a decreased ability to perform daily tasks. Previously, we have reported that each macular layer was significantly correlated with SAP measurements in relatively younger glaucoma patients (median age of 65.7 years), and used data from macular OCT parameters to successfully create a simulated visual field [20]. In the present study, we found that even in older individuals, OCT parameters, which are structural measurements that do not depend on patient responses, correlated well to the SAP measurements after the reliability of VF test results and OCT measurements was ensured. The structure/function correlation found by our study was comparable to that found by previous studies of younger patients (Fig 7). This indicates that there may be the considerable possibility of objective and quantitative method, such as OCT, as an assessing tool for glaucoma diagnosis and progression in older patients with glaucoma, when VF examinations are not possible means.

We acknowledge several limitations to our study. First, it was an institution-based cross-sectional study, a design with intrinsic drawbacks due to the older age of the study subjects. All of the participants were of Japanese ethnicity, and a large proportion was female, who live longer than male in Japan. The RNFL is generally significantly thicker in women than in men. Additionally, patients with normal-tension glaucoma were included in the POAG group due to the high prevalence of normal-tension glaucoma in Japan. These patients might have had different characteristics than those with high-tension glaucoma [54], [55]. However, these characteristics of our study mean that the present results will be especially useful in Asian countries, where the prevalence of normal-tension glaucoma is high. A second limitation of our study is that our findings on the correlation between the structure and function of the eye may have been influenced by age, sex, axial length, glaucoma severity, and myopia, while our findings on macular sensitivity may have been affected by cataracts, which are often co-morbid with glaucoma. After the exclusion from the study of patients with high myopia (defined as an axial length of more than 26 mm) to limit the influence of this factor, all of the remaining subjects meeting the eligibility criteria had pseudophakic eyes. It has been reported that cataracts can cause the Cirrus and Stratus OCT devices to significantly underestimate RNFLT [56], although OCT measurements of cpRNFLT have also been shown to be very reproducible in pseudophakic eyes, to the same degree as in eyes with clear media [57]. However, not all older patients with glaucoma have pseudophakic eyes. Thus, when interpreting the results of the present study, factors related to the lenses of the subjects should be carefully considered. In addition, it has been reported that a residual amount of RNFL thickness remains even in OCT measurements of patients with advanced glaucoma [3]. This phenomenon is called the floor effect, and may have affected our analysis of the structure/function relationship. Lastly, another limitation of the present study was the small number of subjects it included, particularly the small number of PXG patients. This may have affected the statistical power of the study to detect significant differences. In the future, a larger, multi-center study may be necessary to establish normative data for older individuals.

## Conclusions

We found that there was a significant correlation between the structure and function of the eye even in older adults with glaucoma. GCCT was the most useful parameter to evaluate the structure/function relationship within the central 10° of the macula in glaucoma eyes, and adjustment for RGC displacement was essential within the central macula. We hope that the results of the present study will encourage the development of an objective and quantitative method of assessing glaucoma in older individuals, which would be a great help for older patients who may have difficulty with VF examinations.
